# Biological Effects of Clinically Relevant CoCr Nanoparticles in the Dura Mater: An Organ Culture Study

**DOI:** 10.3390/nano4020485

**Published:** 2014-06-16

**Authors:** Iraklis Papageorgiou, Thomas Abberton, Martin Fuller, Joanne L. Tipper, John Fisher, Eileen Ingham

**Affiliations:** 1 IMBE (Institute of Medical & Biological Engineering), School of Biomedical Sciences, Faculty of Biological Sciences, University of Leeds, Leeds LS2 9JT, UK; E-Mails: thomasabberton@gmail.com (T.A.); M.J.G.Fuller@leeds.ac.uk (M.F.); J.L.Tipper@leeds.ac.uk (J.L.T.); E.Ingham@leeds.ac.uk (E.I.); 2 IMBE, School of Mechanical Engineering, University of Leeds, Leeds LS2 9JT, UK; E-Mail: J.Fisher@leeds.ac.uk

**Keywords:** total disc replacement, meninges, dura mater, nanoparticles, immunohistochemistry, cytokines, matrix metalloproteinases

## Abstract

Medical interventions for the treatment of spinal disc degeneration include total disc replacement and fusion devices. There are, however, concerns regarding the generation of wear particles by these devices, the majority of which are in the nanometre sized range with the potential to cause adverse biological effects in the surrounding tissues. The aims of this study were to develop an organ culture model of the porcine dura mater and to investigate the biological effects of CoCr nanoparticles in this model. A range of histological techniques were used to analyse the structure of the tissue in the organ culture. The biological effects of the CoCr wear particles and the subsequent structural changes were assessed using tissue viability assays, cytokine assays, histology, immunohistochemistry, and TEM imaging. The physiological structure of the dura mater remained unchanged during the seven days of *in vitro* culture. There was no significant loss of cell viability. After exposure of the organ culture to CoCr nanoparticles, there was significant loosening of the epithelial layer, as well as the underlying collagen matrix. TEM imaging confirmed these structural alterations. These structural alterations were attributed to the production of MMP-1, -3, -9, -13, and TIMP-1. ELISA analysis revealed that there was significant release of cytokines including IL-8, IL-6, TNF-α, ECP and also the matrix protein, tenascin-C. This study suggested that CoCr nanoparticles did not cause cytotoxicity in the dura mater but they caused significant alterations to its structural integrity that could lead to significant secondary effects due to nanoparticle penetration, such as inflammation to the local neural tissue.

## 1. Introduction

Lower back and neck pain are major health issues in Western countries [1]. A variety of pathologies can cause low and high back pain including degenerative disc disease. Degenerative disc disease results in abnormal motion leading to biomechanical instability and recurrent pain. When conservative treatment fails, patients and doctors consider other options, such as surgery. For many years, spinal fusion has been the gold standard of surgical treatment but the long term results have been poor and complications are common [2]. An alternative surgical procedure involves the replacement of the intervertebral disc with an artificial disc. Artificial discs include nuclear spacers (e.g., Fernstrom endoprosthesis, Ray prosthetic disc nucleus, Zimmer PEEK Ardis Interbody Inserter), total disc replacement devices (e.g., Charite, ProDisc, Kineflex, Cervicore, Maverick) and elastomeric devices (AcroFlex, Physio-L) [3,4]. All of these devices restore and maintain spinal segment motion while relieving pain. There are, however, concerns regarding total disc replacements in relation to the generation of wear debris that could lead to osteolysis, as well as other local and systemic effects [1].

A study of the AcroFlex device has highlighted the potential problem of wear particles leading to osteolysis [4]. Recently, the US Food and Drug Administration (FDA) issued a recall on the PEEK Ardis Interbody spacer due to the risk of fragmentation potentially leading to tears in the dura mater, leakage of cerebrospinal fluid and nerve injury [5]. Patients with metal-on-metal (MoM) total disc replacements have increased serum metal ion concentrations following surgery [6]. Several case reports have also revealed that MoM disc replacement devices can lead to the formation of granulomatous tissue with significant levels of metalosis that may occlude the surrounding arteries and veins and potentially cause spinal stenosis through infiltration of the spinal canal [7,8,9]. One case report documented signs of chronic inflammation in the epidural space during revision surgery of a cervical disc replacement. Histopathological analysis revealed a lymphocyte-dominated response that was similar to the soft tissue reaction reported in some patients with failed MoM hip prosthesis [10]. This is of particular concern for MoM devices, given the potential for toxicity of metal wear particles and the proximity to the spinal cord.

Anatomically, the meninges enclose the spinal cord, and they consist of an outer fibroelastic layer, the dura mater, an intermediate arachnoid mater and the innermost pia mater [11]. The dura mater acts as a protective membrane for the spinal cord. It prevents the infiltration of foreign bodies harmful to the neural tissue and can protect the spinal cord from mechanical stresses such as stretching during movement and from changes in cerebrospinal fluid pressure [12,13,14].

The biological effects of wear particles generated in TDRs and their potential interactions with the dura mater (meninges) have the potential to pose a significant clinical problem, as evidenced by reports on adverse soft tissue reactions to wear products in patients [10]. There are a limited number of studies that have investigated how particles interact with the dura mater and ultimately the central nervous system [15,16]. In our previous study [17], porcine fibroblasts and epithelial cells isolated from the dura mater were treated with clinically-relevant CoCr nanoparticles. The CoCr particles significantly reduced the viability of the dural epithelial cells in a dose-dependent manner and the cells secreted significant amounts of IL-8 at the highest particle doses [17]. Since the isolated cells grown in monolayer lack three-dimensional fibroblast/epithelial cell-cross-talk and cell/matrix interactions [18] it was important to extend our studies from a cellular level to a more physiological model of the intact tissue and to utilise multiple approaches to assess the overall biological response [19].

This study was therefore designed to test the hypothesis that CoCr nanoparticles cause significant biological effects on the dura mater. In order to test this hypothesis, an aseptic organ-culture model of the porcine dura mater was developed and exposed to different doses of 20–60 nm sized CoCr nanoparticles. The biological effects of the CoCr treatment and the structural changes to the tissue were assessed using tissue viability assays, assays of cytokine production, histology, immunohistochemistry, and TEM imaging of the treated *vs.* untreated tissue.

## 2. Results and Discussion

### 2.1. Histological Characterisation and Viability Assessment of Dura Mater in Organ Culture

**Figure 1:**
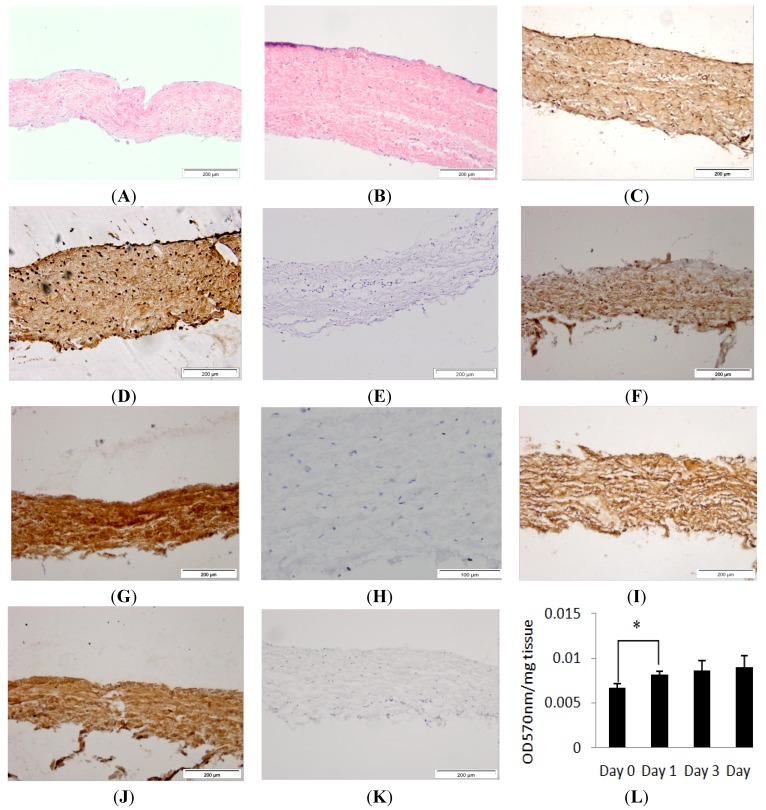
Images of histological sections of the isolated porcine dura-mater tissue at Day 0 (**A**) and after seven days in organ culture (**B**) stained with H&E. Immunohistochemical staining of the porcine dura mater with antibodies to fibronectin (**C**,**D**), collagen I (**F**,**G**), collagen II (**I**,**J**) at Day 0 and Day 7 respectively. Isotypes controls for fibronectin (**E**), collagen I (**H**) and collagen II (**K**) antibodies. Tissue viability over a period of 7 days in organ culture as determined by MTT assay (**L**). Results are presented as OD at 570 nm per mg of wet weight of tissue. Data is presented as the mean (*n* = 3) ± 95% confidence limits. Data were analysed by one-way analysis of variance and individual differences between group means determined by the T-method. * indicates significant difference (* *p* < 0.05).

Porcine dura mater, freshly isolated from the meninges surrounding the spinal cord was processed for histology and sections were stained using H & E (Hematoxylin & Eosin) and visualised by light microscopy to determine its native characteristics. Native porcine dura mater was composed of an outer epithelial cell layer and an underlying dense collagen I and II matrix with interspersed fibroblast cells (Figure 1A,C,E,G). A more comprehensive study of the composition of the porcine dura mater has been reported in a previous study by Papageorgiou *et al.* [14]. The structural arrangement agreed with an ultrastructural study of the human meninges that showed that the dura mater was composed of an outermost loosely arranged fibroelastic layer, a central fibrous layer and an innermost cellular layer [11].

Porcine dura mater, freshly isolated from the meninges surrounding the spinal cord was maintained in organ culture for seven days and then subject to histological and immunohistochemical evaluation. After seven days of *in vitro* culture, the histological characteristics of the porcine dura mater remained unchanged (Figure 1B,D,F,H). There was no alteration in the structure of the dura mater. Porcine dura mater maintained in organ culture over a seven day period was also assessed for tissue viability using MTT (3-(4,5-dimethylthiazol-2-yl)-2,5-diphenyltetrazolium bromide) assay and there was no loss of viability (Figure 1K). There was a significant (*p* < 0.05) increase in MTT conversion after the first day of organ culture which was most probably due to the recovery of the tissue after the initial dissection process. MTT assay and immunohistochemical staining, have been used previously to assess tissue viability and tissue morphology in placental tissue explant cultures and buccal mucosa [20,21].

**Figure 2:**
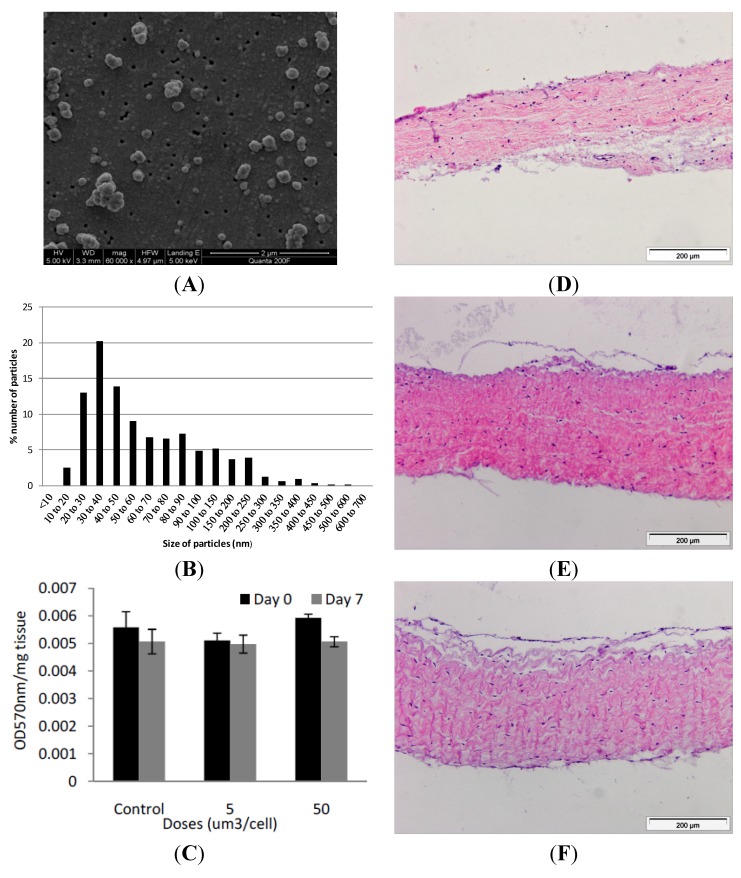
(**A**) Representative image of cobalt-chrome nanoparticles that were generated using a pin-on-plate tribometer. Images were captured by FEGSEM; (**B**) Size distribution of cobalt-chrome nanoparticles. The size distribution of the cobalt-chrome nanoparticles was determined from SEM images taken from different locations on the filter membrane and analysed using Image Pro-Plus imaging software; (**C**) Effects of CoCr nanoparticles on the viability of dura-mater at 0 and 7 days of culture. Porcine dura mater tissue was cultured in the absence (control) and presence of CoCr nanoparticles at an estimated dose of 5 and 50 µm^3^ per epithelial cell and viability determined by the MTT assay. Data are expressed as the mean (*n* = 3) ± 95% confidence limits. The data were analysed by ANOVA which revealed no significant variation between control and CoCr-treated tissues at day 0 or day 7. Images of dura mater tissue exposed to cobalt-chrome nanoparticles at an estimated dosage of 0 (**D**), 5 (**E**) and 50 (**F**) µm^3^ per epithelial cell for a period of 7 days and stained with H&E. Tissues sections shown are orientated so that the outer epithelial layer is closest to the top of the section.

### 2.2. Cobalt-Chrome Alloy Nanoparticle Generation and Characterisation

Nanometre-sized cobalt-chrome particles were generated using a pin-on-plate tribometer in distilled water and characterised using field emission gun scanning electron microscopy (FEGSEM) for size and shape. Six batches of particles were prepared and pooled. The particles were sonicated for 10 min prior to filtration to disperse agglomerates. The particles were round or oval in shape, with a few showing jagged edges (Figure 2A). Utilising Image Pro-Plus image analysis software, it was found that 68% of the particles were less than 100 nm in size, with 100 particles analysed from 6 randomly chosen regions of the filter. The highest percentile of nanoparticles fell within the 30–40 nm sized range (Figure 2B). Additional information regarding the properties of these CoCr nanoparticles, such as the sedimentation rate in tissue culture medium and metal ion release can be found in our previous publication [17].

### 2.3. Exposure of the Porcine Dura Mater to Cobalt-Chrome Nanoparticles

Samples of porcine dura-mater tissue were incubated with CoCr nanoparticles at an estimated dose of 0, 5 and 50 µm^3^∙cell^−1^ (0, 0.833 and 8.33 µg∙mm^−3^ of tissue). Despite sonication of the particles immediately prior to tissue exposure, re-agglomeration of the particles was unavoidable [22,23]. Particle agglomeration would also likely occur *in vivo*. All exposures were repeated in triplicate at each time point and at each dose. The effect of nanoparticle exposure on tissue viability was determined immediately following exposure (day 0 of exposure) and after seven days of exposure (day 7) by MTT assay (Figure 2C). Optical densities for the MTT assays were corrected against tissue only controls without MTT. At day 0, exposure of the dura mater to 5 or 50 µm^3^∙cell^−1^ of CoCr particles had no significant effect on tissue viability (*p* > 0.05) compared to the control tissue which had not been exposed to particles. After seven days of exposure to CoCr nanoparticles at 5 or 50 µm^3^∙cell^−1^, there was no significant difference in tissue viability compared to the control tissues. Both the day 0 and day 7 tissue samples across control and experimental groups showed consistent levels of viability (0.005–0.006 OD 570 nm∙mg^−1^ tissue) and there were no significant differences between any groups (*p* > 0.05).

The results for tissue viability following CoCr nanoparticle exposure were not entirely inconsistent with the results obtained in our previous study in which dural epithelial cells, but not dural fibroblasts isolated from the porcine dura mater showed a reduction in viability following exposure to CoCr nanoparticles at the same doses [17]. The differences between the results of the toxicity of CoCr nanoparticles obtained in organ culture and monolayer cell culture may be explained by differences in the behaviour of the epithelial cells in the two systems. With some toxic chemicals, the functional status of the cell (intrinsic cell characteristics) rather than the cell type determines the extent of its sensitivity to chemical toxicity. Such cellular characteristics may include membrane binding and permeability, intracellular synthetic pathways and adaptive and recovery mechanisms, which may differ in organ culture and monolayer cell culture [24]. Alternatively the results may simply have been due to the fibroblasts, being the predominant cell type in the dura, contributing the majority of MTT conversion in the assay of the tissue, with the assay not being sensitive enough to detect toxic effects of the particles on the epithelial cells. Further studies using live/dead cell staining to determine any differential effects of the CoCr nanoparticles on the epithelial cells and fibroblasts in the organ culture would provide further insight.

When the dura-mater tissue was cultured for seven days with 5 µm^3^ per epithelial cell of CoCr nanoparticles there were structural distortions to the epithelial layer compared to the control tissue observed in histological sections (Figure 2D,E). The epithelial layer detached from the outer edges of the tissue across an estimated 40% of the tissue sections. The remainder of the tissue appeared similar in structure to control tissues. Porcine dura-mater tissue incubated for seven days with 50 µm^3^∙cell^−1^ of CoCr nanoparticles showed similar loosening of the collagen layer near the epithelium as well as the detachment of epithelial layer across the majority of the tissue section (Figure 2F). H & E staining of tissue sections also showed some loosening of the collagen layers immediately adjacent to the detached epithelial layer in small areas across the tissue in all replicates.

**Figure 3:**
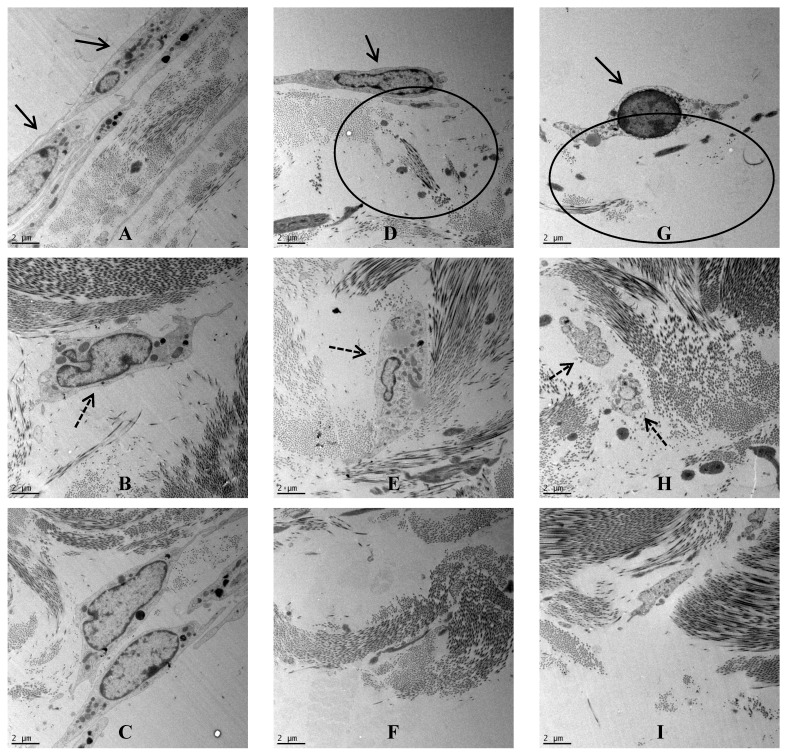
TEM images across the dura mater tissue that was exposed to CoCr nanoparticlesfor a period of seven days. (**A**–**C**) Images of the control dura mater from the epithelial region (**A**), inner collagen region (**B**) and the basal side of the dura mater close to arachnoid mater (**C**); (**D**–**F**) Images of the dura mater exposed to an estimated dose of 5 µm^3^ of CoCr particles per epithelial cell, from the epithelial region (**D**), inner collagen region (**E**), and the basal side of the dura mater close to arachnoid mater (**F**); (**G**–**I**) Images of the dura mater exposed to an estimated dose of 50 µm^3^ of CoCr particles per epithelial cell, from the epithelial region (**G**), inner collagen region (**H**), and the basal side of the dura mater close to arachnoid mater (**I**). Single continuous black arrows indicate the dural epithelial cells. Single dotted black arrow indicated the dural fibroblast cells. Black circles indicated disruption of the collagen layer.

Porcine dura-mater was visualised using transmission electron microscopy to determine the ultrastructural characteristics of the native tissue and to determine any possible structural defects following exposure to nanoparticles. In the control tissue after 7 days of organ culture, the epithelial cell-layer was observed surrounding the dura-mater that was mainly composed of a network of highly ordered collagen fibers (Figure 3A). Interspersed with the collagen bundles were regularly arranged fibroblasts (Figure 3B). This tight structure appeared to be disrupted when the dura mater was exposed to CoCr nanoparticles for 7 days. In the low dose organ culture (5 µm^3^∙cell^−1^), the epithelial layer was disrupted on the apical side (Figure 3D), the fibroblasts appeared vacuolated (Figure 3E) and the basal layer of collagen appeared loosened (Figure 3F). Similar structural changes were observed in the tissues treated with the highest dose of CoCr particles (50 µm^3^∙cell^−1^). In addition to disruption at the surface, the collagen fibres on the apical side were also disrupted (Figure 3G). The fibroblasts deep in the dura mater also showed signs of cellular degradation (Figure 3H), and in addition the basal layer of collagen also appeared loosened (Figure 3I).

These preliminary histological and TEM observations suggested that there were structural alterations in the dura mater following exposure to CoCr nanoparticles for a period of seven days. In future studies it will be important to quantify this phenomenon further for example by: measurement of the transepithelial electrical resistance of the membrane, assessment of epithelial integrity through ZO-1 and E-cadherin staining, determination of total collagen and glycosaminoglycan content and biomechanical tests to determine any functional changes in the properties of the dura mater. In addition, the orientation of the collagen fibers could be quantified using atomic force microscopy [25] to determine any effects of CoCr nanoparticle exposure on the packing of the collagen fibers.

**Figure 4:**
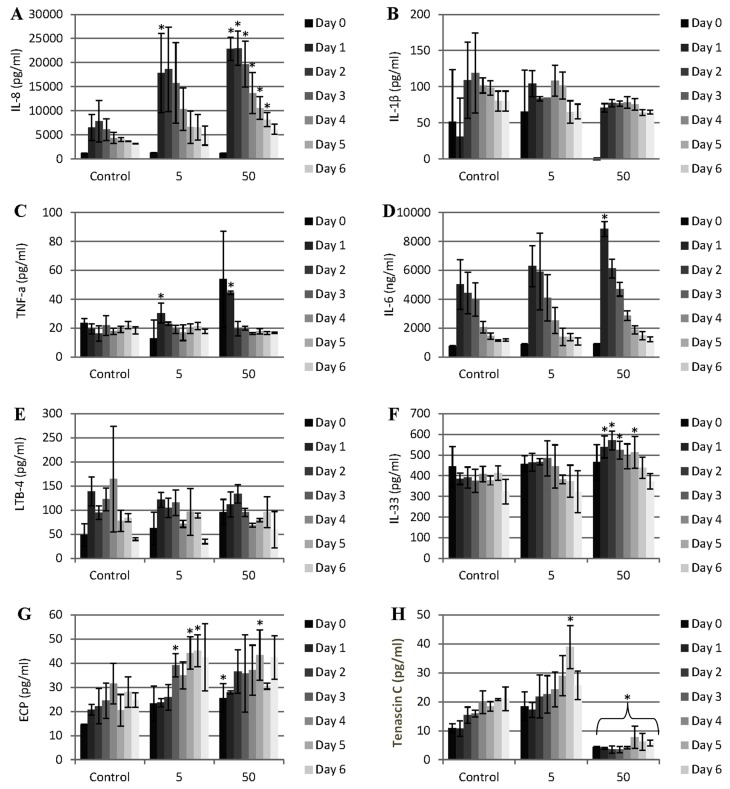
Pattern of cytokine and other mediator release during the exposure of porcine dura mater to CoCr nanoparticles for a period of 0–7 days. The dura mater organ culture was exposed to estimated doses of 0 (control), 5 and 50 µm^3^ of CoCr nanoparticles per epithelial cell. The factors that were investigated were IL-8 (**A**), IL-1β (**B**), TNF-α (**C**), IL-6 (**D**), LBT-4 (leukotriene B4) (**E**), IL-33 (**F**), ECP (eosinophil chemotactic protein, eotaxin, CCL-11) (**G**), and tenascin C (**H**). Data is expressed as the mean (*n* = 3) ±95% confidence limits. Data were analysed by one-way analysis of variance and individual differences between group means determined by the T-method. * indicates significantly difference between control and CoCr-treated tissue (* *p* < 0.05).

### 2.4. Proinflammatory Cytokine Response of the Dura Mater Exposed to Cobalt-Chrome Nanoparticles

In order to determine if the CoCr nanoparticles had any effect on pro-inflammatory mediator release from the dura mater, the release of interleukin 8 (IL-8), tumour necrosis factor-α (TNF-α), IL-6, IL-1β, and leukotriene B-4 (LTB-4) was measured daily in the organ culture conditioned medium for a period of seven days after the exposure of the organ culture to 5 and 50 µm^3^ CoCr nanoparticles per epithelial cell. There was significant release of IL-8 associated with both doses of CoCr nanoparticles compared to the control (Figure 4A). At the highest particle dose, the pattern of IL-8 release was continuous for the whole period of treatment (seven days). This IL-8 release was in agreement with the results of the previous study by Behl *et al.* [17], in which it was shown that dural fibroblasts and dural epithelial cells exposed to CoCr nanoparticles released significant amounts of IL-8. IL-8 release is a sign of a pro-inflammatory response of the tissue. IL-8 has a role in the recruitment and activation of neutrophils and T-lymphocytes [26,27]. In addition to its action as a chemokine, IL-8 induces neutrophils to release lysosomal enzymes and down-regulates collagen expression by human fibroblasts [26]. It might be hypothesized that *in vivo*, exposure of the dura mater to CoCr nanoparticles could stimulate the release of IL-8 resulting in the recruitment of neutrophils and release of lysosomal enzymes that would alter the structural integrity of the dura mater.

The pattern of TNF-α and IL-6 release was transient (Figure 4C,D). TNF-α was released only during the first 24 h of exposure to nanoparticles in a dose dependent manner. According to Harada *et al.* [26] the production of IL-8 occurs in the presence of inflammatory stimuli such as IL-1 and TNF-α. Hence, IL-8 release could have been initiated by the transient TNF-α response of the tissue. IL-6 was also was significantly elevated in the first day of exposure to CoCr nanoparticle but only at the highest particle dose (50 µm^3^∙cell^−1^). IL-6 release from the organ cultures exposed to CoCr nanoparticles was not consistent with the results from our previous study in which no significant IL-6 release was observed over a period of 4 days at a range of particle doses for both dural fibroblasts and dural epithelial cells [17]. Similar results were also observed in a study by Papageorgiou *et al.* [28], in which exposure of dermal fibroblasts to CoCr particles did not cause the release of significantly elevated levels of IL-6. The IL-6 release that was observed in the organ culture exposed to CoCr nanoparticles could have been the result of intercellular signaling between dural epithelial cells and dural fibroblasts. Such epithelial cell/fibroblast interactions have been documented in the lung airway wall [29] and involves cytokines such as IL-6 which can lead to [30] remodeling of the airway wall. Similarly, it can be hypothesized that the dural epithelial layer activates the underlying fibroblasts which in turns activate the epithelial layer further leading to an enhanced cytokine response.

There was no IL-1β or LTB-4 (leukotriene B4) released at any time or dose of exposure to the nanoparticles (Figure 4B,E).

IL-33 is a member of the IL-1 cytokine family and is released by stressed epithelial cells and fibroblasts. Significant amounts of IL-33 were released between day 2 and day 5 at the highest CoCr nanoparticle treatment dose of 50 µm^3^∙cell^−1^ (Figure 4F). This cytokine specifically modulates Th2-type proinflamatory signals after its release from necrotic cells that may promote eosinophilia [31]. A similar phenomenon occurred with ECP release at the low particle treatment dose of 5 µm^3^∙cell^−1^ (Figure 4G). Nearly double the amount of ECP, relative to the control levels, was released from the treated dura mater between day 3 and day 6 of exposure to CoCr nanoparticles. Since ECP is documented to promote eosinophilia [31], it might be hypothesized that CoCr nanoparticle exposure of the dura mater *in vivo* could stimulate the release of IL-33 and ECP that would in turn promote eosinophil recruitment to the site of CoCr nanoparticle exposure.

Tenascin C in an extracellular matrix glycoprotein that has been reported to be expressed in response to tissue injury and infection [32,33]. Its expression is linked with cytokines associated with inflammation such as TNF-α [34]. There was a significant release of tenascin C when the organ culture was exposed to the low particle dose (5 µm^3^∙cell^−1^) after six days. However, for the high particle dose (50 µm^3^∙cell^−1^) significantly lower amounts of tenascin C were released at all-time points, compared to the control values. It is possible that TNF-α release in response to the low dose of CoCr nanoparticles caused the induction the tenascin expression. However, at the high dose of CoCr nanoparticles the drop of tenanscin C expression (even below the control tissue levels) could be attributed to the activation of MMP’s and the cleavage of tenascin-C. There is a feedback loop between induction of metalloproteases by tenascin-C and cleavage of tenascin-C by these enzymes [32]. Chiquet-Ehrisman *et al.* [35] suggested that the production of tenascin C in inflamed tissues belongs to a set of mechanisms required to control the spread of inflammation.

### 2.5. MMP and TIMP-1 Expression in Dura Mater after Exposure to Cobalt-Chrome Nanoparticles

Generally, MMP’s are not expressed in healthy tissues. However, MMP expression can be detected in inflamed tissues and/or in tissues under repair and remodelling. Metalloproteinases control the degradation of the extracellular matrix (as their name implies), they modulate chemokine activity of the inflamed region and they establish different chemokine gradients to modulate the influx of inflammatory cells [36]. The expression of MMPs was therefore investigated following exposure of the dural tissue in organ culture to CoCr nanoparticles.

The tissue were investigated for the expression of MMP-1, -3, -9, and -13 since these are the MMPs implicated in collagen, and fibronectin degradation as well as disruption of the basement membrane and general remodelling of inflamed tissues [36,37]. The staining pattern of the CoCr-treated tissue is summarised in Table 1.

**Table 1:** Expression pattern of different matrix metalloproteinases in dural epithelial and fibroblast cells as well as in the extracellular matrix exposed to CoCr wear nanoparticles of different doses. Control tissues showed no positive staining and are not included. (Intensity of staining was assessed as: −−−: no staining, +/−: very light staining, +: light staining, ++: strong staining, +++: very strong staining).

	CoCr Nanoparticle Treatment (µm^3^ per epithelial cell)					
	5 µm^3^			50 µm^3^		
Type of MMP/TIMP	*Epithelial cells*	*Fibroblasts*	*Extracellular matrix*	*Epithelial cells*	*Fibroblasts*	*Extracellular matrix*
MMP-1	++	+	−−−	+	+	−−−
MMP-3	++	+	+	+	+/−	−−−
MMP-9	+++	++	++	+++	++	+++
MMP-13	+	+/−	+/−	++	++	++
TIMP-1	+++	+/−	−−−	+	+/−	−−−

**Figure 5:**
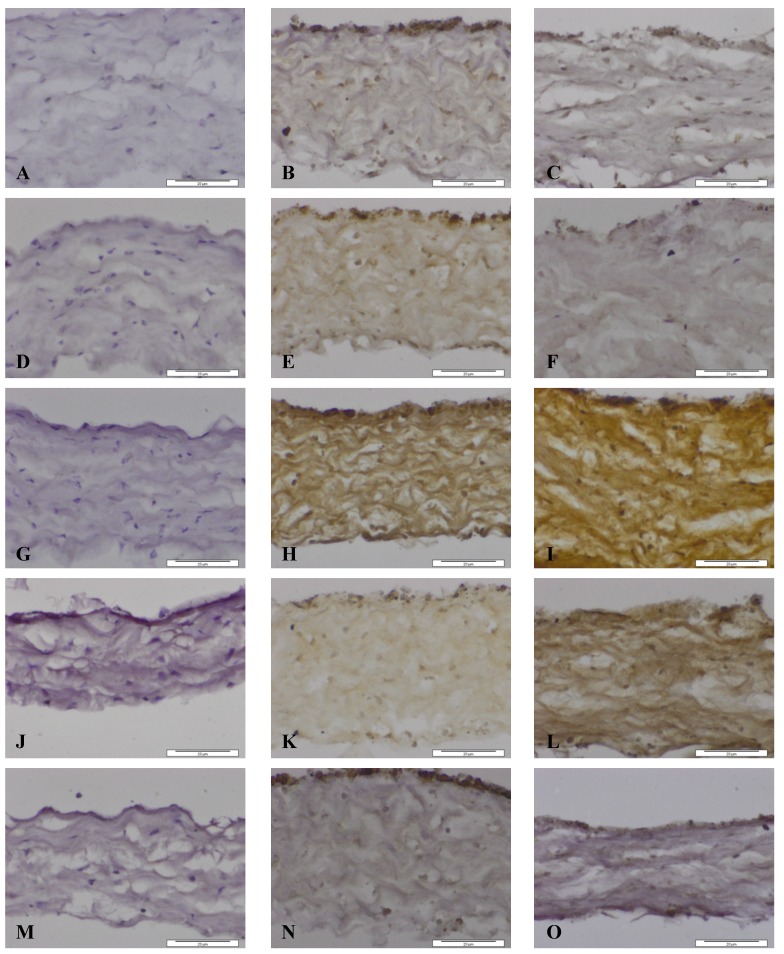
Images of dura mater exposed for seven days to CoCr nanoparticles and stained for the presence of matrix metalloproteinases and TIMP-1 by immunhistochemistry. Images of section of control dura mater tissue (**A**,**D**,**G**,**J**,**M**), dura mater exposed to an estimated dose of 5 µm^3^ CoCr nanoparticles per epithelial cell (**B**,**E**,**H**,**K**,**N**) and dura mater exposed to an estimated dose of 50 µm^3^ CoCr nanoparticles per epithelial cell (**C**,**F**,**I**,**L**,**O**). The tissues were stained for MMP-1 (**A**–**C**), MMP-3 (**D**–**F**), MMP-9 (**G**–**I**), MMP-13 (**J**–**L**), TIMP-1 (**M**–**O**).

When the dura mater was exposed to both doses of nanoparticles MMP-1 was expressed in the cytoplasm of both dural epithelial and dural fibroblast cells (Figure 5B,C). The pattern of staining of MMP-3 was, however different. At the low particle dose (5 µm^3^∙cell^−1^) MMP 3 was expressed by both cell types and was also present in the collagen region (Figure 5E). At the high particle dose (50 µm^3^∙cell^−1^), there was only a limited amount of staining of the epithelial cells, but no staining was observed in dural fibroblasts or the collagen matrix (Figure 5F). According to a study by Chao *et al.* [38] increased levels of eotaxin not only induce MMP-3 gene expression but also promote MMP-3 protein secretion. It may be hypothesized that since eotaxin was released from the tissue during the six days of exposure to the low dose CoCr nanoparticles (Figure 4G), MMP-3 was produced and secreted from epithelial and fibroblast cells in response to eotaxin.

MMP-9 was present in both dural epithelial and fibroblast cells as well as in the collagen region of the dural tissue that had been exposed to both doses of CoCr nanoparticles (Figure 5H,I). MMP-9 is reported to digest gelatin [37] and it may be hypothesized that it contributes to the structural alterations observed in CoCr-treated dura mater.

Epithelial and fibroblast cells were stained positively for MMP-13 (Figure 5J,L), however, with the low dose treatment (5 µm^3^∙cell^−1^) the collagen layer did not stain as intensively as with the high dose treatment (50 µm^3^∙cell^−1^). This metalloproteinase, cleaves collagen I, II, and III [36] and may have contributed to the structural loosening that was observed in the dura mater treated with CoCr nanoparticles.

MMP activities in tissues are regulated by specific inhibitors such as the tissue inhibitors of metalloproteinases (TIMPs). Four TIMP’s (TIMP-1, -2, -3, -4) have been identified in vertebrates and their expression is regulated in tissue remodelling [37]. TIMP-1 was chosen for investigation because it is a stromal factor that has a wide spectrum of functions in different tissues and can inhibit all the MMP’s investigated above [37,39]. The pattern of staining of TIMP-1 is shown in Figure 5M–O. At both particle treatment doses the dural epithelial cells were stained intensively; whereas the dural fibroblast cells and the collagen layer around them did not have any evidence of staining. Since the principal physiological role of TIMP-1 is to regulate the degradation of the basement membrane and the extracellular matrix by MMPs [40], the presence of TIMP-1 in the dural epithelial cells might indicate that this was upregulated in order to limit tissue degradation. It will be important to determine in future studies whether the observed MMP expression in the dura mater following exposure to CoCr nanoparticles correlates with enzyme activity using zymography [41].

## 3. Experimental Section

### 3.1. Aseptic Isolation of the Dura Mater from Pigs

Three pigs from the University of Leeds farm (Large White females, 65 kg) were humanely killed using a UK Home Office procedure and were brought to the dissection table within minutes of death. The skin on the back was swabbed with iodine solution and the vertebral column was aseptically dissected and transferred to the laboratory for further aseptic dissection. The vertebrae and spinal canal was dissected with bone cutters revealing the spinal cord with the surrounding meninges. The extracted spinal cord from each animal with the meninges still attached, was placed in an antimicrobial solution (200 mL) of Gentamicin (Sigma, Dorset, UK) 50 ng∙mL^−1^ and Nystatin (Sigma, Dorset, UK) 100 U∙mL^−1^ in medium 199 (Sigma, Dorset, UK) supplemented with foetal bovine serum (20% (*v*/*v*), Lonza, Slough, UK), L-glutamine (2 mM, Lonza, Slough, UK), sodium pyruvate (1.1 mg∙mL^−1^, Sigma, Dorset, UK), heparin (10 U∙mL^−1^), penicillin/streptomycin (50 U∙mL^−1^ and 50 mg∙mL^−1^ respectively, Lonza, Slough, UK) and endothelial growth factor (15 µg∙mL^−1^, Sigma, Dorset, UK) for an hour at room temperature.

### 3.2. Tissue Processing for Histology and H & E Staining

Dura-mater tissue specimens were placed in zinc fixative solution (0.05% *w*/*v* calcium acetate, 0.5% *w*/*v* zinc acetate, 0.5% *w*/*v* zinc chloride in 0.1 M Tris) for 24 h at room temperature and then cut into 1 cm wide blocks, which were processed in plastic cassettes (Histosette) into paraffin wax (Bios Europe Ltd., Skelmersdale, UK) using an automated tissue processor (Leica TP 1020, Leica Microsystems, Newcastle upon Tyne, UK).

A microtome (RM2125RTR, Leica Microsystems, Newcastle upon Tyne, UK) was used to section the wax embedded blocks into 4–10 µm thick sections and the sections were then transferred onto Superfrost Plus slides (VWR International, Lutterworth, UK). The sections were air-dried for 30 min, dewaxed and rehydrated. Sections were stained using Mayer’s haematoxylin (Bios Europe Ltd., Skelmersdale, UK) and eosin (Bios Europe Ltd., Skelmersdale, UK) using a standard method, dehydrated and mounted using DPX mountant (Bios Europe Ltd., Skelmersdale, UK) Images were captured using an upright microscope (Olympus BX51, Southend-on-Sea, UK), a digital camera (Olympus XC50, Southend-on-Sea, UK) and Cell^B Image analysis software (Olympus, UK, Southend-on-Sea, UK).

### 3.3. Immunohistochemistry

Porcine dura mater tissue was characterised using immunohistochemistry with antibodies specific for collagen II (mouse anti-human polyclonal, COL-II, mouse IgG1; 1:50 dilution; Millipore Limited, Watford, UK), collagen I (mouse anti-human monoclonal, mouse IgG1k; 1:50 dilution; Millipore Limited, Watford, UK) and fibronectin (rabbit anti-human polyclonal, rabbit immunoglobulin fraction; 1:400 dilution, DakoCytomation, Cambridgeshire, UK).

Sections were subject to antigen retrieval using proteinase K (DAKO, S3020) for 20 min prior to immunohistochemical staining using the Ultra vision kit (Envision, Birmingham, UK) and counterstaining with haematoxylin as described previously [14] Images were captured as above.

### 3.4. CoCr Nanoparticle Production and Characterisation

Particles were generated in a six station pin-on-plate tribometer (manufactured in the School of Mechanical Engineering, Leeds University, UK) with water as the lubricant under a load of 80 N for 40 h as described previously [14,17]. The pins and plates were manufactured from medical grade wrought high carbon (<0.2%) CoCr alloy ASTM F1537 with smooth counterfaces (Ra: 0.01–0.02 µm). Wear particles were sterilised at 180 °C for 4 h and their mass determined by gravimetric analysis. The particles were suspended at 1 mg∙mL^−1^ in sterile water. A sample of the particle suspension was captured on a 0.015 µm polycarbonate filter membrane (Millipore Limited, Watford, UK) and characterized using field emission gun scanning electron microscopy (FEGSEM; FEI, Eindhoven, The Netherlands) and Image Pro-Plus^®^ image analysis software (Media Cybernetics, Rockville, MD, USA). A total of 100 particles per image were analysed from 3 images.

### 3.5. Maintenance and Treatment of Dura Mater with CoCr Nanoparticles

The dissected spinal cord with the meninges was aseptically dissected into 12 mm^2^ segments and the dura mater was removed and placed on top of sterile Transwell^®^ permeable supports/inserts (diameter of pores: 3 µm; Corning, Flintshire, UK). The orientation of the dura mater was arranged with the epithelial layer was facing upwards. The inserts with the dura mater were then placed in a 6-well plate and the wells filled with m199 medium (Sigma, Dorset, UK) supplemented with foetal bovine serum (20% (*v*/*v*), Lonza, Slough, UK), L-glutamine (2 mM, Lonza, Slough, UK), sodium pyruvate (1.1 mg∙mL^−1^, Sigma, Dorset, UK), heparin (10 U∙mL^−1^), penicillin/streptomycin (50 U∙mL^−1^ and 50 mg∙mL^−1^, Lonza, Slough, UK) and endothelial growth factor (15 µg∙mL^−1^, Sigma, Dorset, UK). The porous nature of the insert ensured that nutrients passed to the tissue. The medium was replaced daily and the conditioned medium stored frozen at −20 °C for mediator assay. The dura mater was hydrated from the epithelial side with a few drops of medium on a daily basis.

The stock suspension of the CoCr nanoparticles (1 mg∙mL^−1^) was diluted 100 and 1000 times in complete medium m199 to achieve particle suspensions of 100 and 10 µg∙mL^−1^ (at an estimated dose of 50 µm^3^ and 5 µm^3^ per cell and replicated three times). The particles were sonicated in an ultrasonicating bath (Grant Instruments Limited, Cambridgeshire, UK) for 10 min immediately before use. The dura mater tissue segments were then treated on the epithelial surface with 1 mL of the appropriate CoCr particle suspensions (100 and 10 µg per tissue section). The particles were added to the epithelial side of the dura mater to mimic the way that the particles would come into contact with meninges *in vivo*. A control sample with 1 mL medium only was also included (negative control). For each treatment three replicate samples were prepared. The samples were incubated for 0 and 7 days in 5% (*v*/*v*) CO_2_ in air at 37 °C.

### 3.6. Determination of the Effects of Cobalt-Chromium Nanoparticles on Tissue Viability

The effects of CoCr nanoparticles on the viability of the dura mater were evaluated using the MTT (3-(4,5-dimethylthiazol-2-yl)-2,5-diphenyltetrazolium bromide) assay according to a standard procedure [42]). The dura mater was dissected aseptically and tissue sections of different weights were incubated with agitation in 600 µL of MTT solution (2.5 mg∙mL^−1^ in PBS) at 37 °C for 4 h. Following incubation, the samples were centrifuged at 300 g for 5 min and the supernatant discarded. DMSO (1 mL) was added and the samples were incubated at 37 °C with rotation overnight (~16 h) in the dark. The samples were centrifuged at 300 g for 5 min and 100 µL aliquots of each sample were transferred to individual wells of a 96-well plate. The optical densities were measured using a Multiskan Spectrum plate reader at 570 nm with a reference filter at 630 nm (Thermo Labsystems, Cheshire, UK). The optical density of each sample was normalised to the weight of the tissue and the data are presented as OD (570–630) mg^−1^ of tissue.

### 3.7. Determination of the Effects of Cobalt-Chromium Nanoparticles on Cytokine and Other Mediator Secretion

The levels of TNF-α, IL-1β, IL-6, and IL-8 present in the conditioned medium collected every 24 h were evaluated using commercial sandwich enzyme-linked immunosorbent assay (ELISA) kits (R & D systems, Abington, UK) following the manufacturer’s instructions. The levels of leukotriene B4 (LTB4) were evaluated using an LTB4 immunoassay kit (R & D systems, Abington, UK) following the manufacturer’s instructions.

The levels of IL-33 (a member of IL-1 superfamily) and eotaxin or CCL-11 eosinophil chemotactic protein (ECP) produced by the cells were measured with an ELISA kit (Blue Gene, Shanghai, China) according to the manufacturer’s instructions. Tenascin C was measured with an ELISA kit (MyBioSource Inc., San Diego, CA, USA) according to manufacturer’s instructions.

### 3.8. Immunohistochemical Staining of the Tissue for MMPs and TIMP-1 Following Treatment with CoCr Nanoparticles

Porcine dura mater tissue (treated with CoCr nanoparticles and untreated controls) was embedded in OTC matrix (R.A Lamb, East Sussex, UK). Sections were cut using a cryotome (Leica, Milton Keynes, UK) and stored at −20 °C until required. Cryosections were stained using antibodies to tissue inhibitor of metalloproteinase’s 1 (TIMP-1; rabbit anti-human polyclonal, P01033, IgG; 1:133 dilution; Abbiotec, San Diego, CA, USA), matrix metalloproteinase 1 (MMP-1; rabbit anti-human polyclonal, IgG; 1:133 dilution; BioSS, Woburn, MA, USA), MMP-3 (mouse anti-human monoclonal; 55-2A4; IgG1/k; 1:250 dilution; Millipore, Billerica, MA, USA), MMP-9 (mouse anti-human monoclonal; 4A3; IgG1; 1:25 dilution; AbD Serotec, Kidlington, UK) and MMP-13 (rabbit anti-human polyclonal; IgG1; 1:133 dilution; Abbiotec, San Diego, CA, USA). Mouse IgG2 (1:30 dilution; DakoCytomation, Cambridgeshire, UK) and rabbit anti-human immunoglobulin factor (1:750 dilution; DakoCytomation, Cambridgeshire, UK) were used as isotype controls.

Cryosections were stained, visualised and images captured as described above (Section 3.3).

### 3.9. Transmission Electron Microscopy (TEM) of the Treated *vs.* Untreated Tissue

Dura mater samples that had and had not been treated with CoCr nanoparticles were fixed with 2.5% (*v*/*v*) glutaraldehyde in 0.1 M phosphate buffer for 2.5 h. The tissue was then washed twice for 30 min with 0.1 M phosphate buffer followed by fixation with 0.1% (*w*/*v*) osmium tetroxide overnight. The tissue was then washed with 0.1 M phosphate buffer and incubated in an ascending alcohol series of 20%, 40%, 60%, 80%, and twice in 100% for 20 min for each wash. Following this, the tissue was washed twice with propylene oxide for 20 min and placed in a 50%–50% Araldite-propylene oxide mixture overnight, followed by a 75%–25% Araldite-propylene oxide for 7 h. Finally the tissue was placed in neat fresh Araldite for 8 h and transferred to embedding moulds to polymerase overnight at 60 °C.

Sections (70 nm) were cut using a glass blade in an Ultracut TEM microtome and picked up on metal grids. The grids were placed on drops of 8% (*v*/*v*) uranyl acetate with the section side facing down for 2 h at room temperature. The grids were then washed with distilled water and placed on drops of lead citrate in 1 M NaOH for 30 min at room temperature. The grids were washed with 0.1 M NaOH solution and then distilled water.

Images were captured from the grids using a CM10 TEM microscope (Philips, Surrey, UK). The images were taken using a two second exposure time at any given magnification.

### 3.10. Statistical Analysis

Data are presented as the mean and 95% confidence limits. Data were analysed using one way analysis of variance followed by calculation of the minimum significant difference between group means using the student T-test method at *p* = 0.05.

## 4. Conclusions

This study, for the first time established a model organ culture of the porcine dura mater. The model was used to study the effects of nanosized CoCr wear particles. Two physiological doses of CoCr nanoparticles (5 and 50 µm^3^ per epithelial cell) were used to treat the dura mater for a period of seven days with no significant effects on cell viability. However, histological analyses as well as TEM imaging of the CoCr treated tissues revealed dose dependent loosening of the dural epithelial layer as well as the underlying connective tissue layer compared to untreated control tissue.

These structural alterations coincided with the release of significant levels of IL-8, IL-6, TNF-α, as well as ECP and tenascin-C. The pattern of release of these mediators over the seven-day period of treatment with the CoCr nanoparticles, suggested that the CoCr particles stimulated a pro-inflammatory response in the tissue. This inflammatory response coincided with the expression of matrix metalloproteinases MMP-1, -3, -9, and -13 which suggested that a catabolic, matrix degrading response occurred in the tissue. MMP expression supported the histological changes observed in the CoCr-treated tissue. A limitation of this study was that it was based on current understanding of CoCr nanoparticle wear particle generation by MoM articulations *in vitro*. There is a lack of current knowledge regarding whether the CoCr wear particles released by MoM TDRs *in vivo* is of a similar nature.

These findings, however, give an initial insight into how CoCr nanoparticles generated in MoM TDR might interact with the dura mater. The results indicated that the particles have the potential to adversely affect the barrier/protective function of the dura mater and also the meninges. Further research is required to assess the biological consequences of the penetration of CoCr nanoparticles through the dura mater, particularly on the function of the dura mater in terms of barrier function and mechanical integrity.

In addition, it is important to assess if the wear debris from other materials used in the spinal implants e.g., polyethylene, polyether ether ketone (PEEK), stainless steel, titanium, have the ability to cause similar structural and biological changes in the dura mater.
